# Comparing Biologic Factor Composition of Birth Tissue-Derived Allografts and Platelet-Rich Plasma

**DOI:** 10.7759/cureus.90259

**Published:** 2025-08-16

**Authors:** Alberto J Panero, Alexandra E Warrick, Hirotaka Nakagawa, Wyatt Andersen, Alan Hirahara, Fernando Fierro

**Affiliations:** 1 Physical Medicine and Rehabilitation, BIOS Orthopedic Institute, Sacramento, USA; 2 Physical Medicine and Rehabilitation, University of California Davis Medical Center, Sacramento, USA; 3 Orthopedics, Stanford University Medical Center, Redwood City, USA; 4 Orthopedics, Sacramento Orthopedics Center, Sacramento, USA; 5 Stem Cell Program, University of California Davis Medical Center, Sacramento, USA

**Keywords:** allograft, amnion, chorion, prp, wharton’s jelly

## Abstract

Background

Orthobiologic products are used for their regenerative potential, but variability in their composition may impact clinical outcomes. This study examined differences in key biologic factors among various products and preparation methods.

Methodology

Different formulations of platelet-rich plasma (PRP) and birth tissue-derived allografts were analyzed for specific biologic factors using laboratory assays.

Results

PRP demonstrated consistent biologic factor levels across samples, while allograft products exhibited notable variability. One category of birth tissue-derived products contained higher levels of a specific growth factor compared to others.

Conclusions

PRP showed greater consistency compared to allografts, which varied significantly between product lots. Standardization may improve the reliability of orthobiologic therapies.

## Introduction

There is continued interest in orthobiologic products for the treatment of orthopedic conditions. Various allograft preparations derived from birth tissues, including amnion, amniotic fluid, and umbilical cord, are emerging as key therapeutics [1]. Autologous orthobiologics, such as platelet-rich plasma (PRP), have also gained popularity, supported by multiple level one studies demonstrating their safety and efficacy in treating various musculoskeletal disorders [2,3]. Allograft orthobiologic tissues, such as placental tissue-derived products, do not require autologous tissue harvests. These products provide therapeutic options for patients whose comorbidities may preclude them from undergoing autologous treatments.

Many orthobiologic therapies have been centered around mesenchymal stem cells (MSCs), which are considered key repair cells of the musculoskeletal system. While MSCs were initially thought to exert their therapeutic effects primarily through cell replacement and differentiation, growing evidence suggests that their primary mechanism of action is their in vivo secretion of trophic and immunomodulatory factors, which direct the body’s innate regenerative processes [4,5]. Current concepts propose that much of the benefit provided by autologous and allograft orthobiologics stems from their ability to deliver critical cell signaling proteins, including growth factors and cytokines. These cells and proteins work together to stimulate anti-inflammatory and analgesic effects, promote cell proliferation, migration, differentiation, and morphogenesis, and enhance the healing of resident tissues [6-8].

While the potential of birth tissue-derived products is promising, they remain a subject of significant controversy in the United States. Manufacturing processes lack standardization, resulting in high variability in product composition. Although lower-level orthopedic studies often report positive outcomes of birth tissue-derived products in conditions such as osteoarthritis and tendinopathies, robust clinical trial data confirming their safety and efficacy are lacking [9,10]. These products gained early popularity as a potential source of direct delivery of viable MSCs [11]. However, our study on amniotic fluid products (AFPs) revealed that viable MSCs were not present in these products as advertised. Despite this, these products may contain therapeutically valuable biologic factors [12]. To date, no studies have directly compared the quantities of biologic markers among various birth tissue-derived allografts and PRP. The objective of this study was to quantify the levels of key biologic factors in commercially available birth tissue-derived products, including particulate placental products, non-particulate placental products, amniotic fluid-based products, and to compare these levels of key biologic factors to those found in PRP.

## Materials and methods

This study was approved by the University of California, Davis Institutional Review Board. Inclusion criteria for participation in venipuncture for PRP collection followed the American Red Cross blood donation guidelines, including (1) being in good general health and feeling well, (2) weighing 110 pounds or more, and (3) no blood donations within the past 56 days [13]. Exclusion criteria included adults unable to provide consent, individuals under 18 years or over 50 years of age, and members of vulnerable populations such as pregnant women, medical students, and prisoners.

We collected blood from three healthy volunteers via a one-time venipuncture. The blood was centrifuged according to the manufacturer’s recommendations to prepare PRP samples using two different PRP preparation systems (Arthrex Angel and Apex Biologics). Leukocyte and platelet concentrations in whole blood were measured as baseline values and compared to their respective PRP preparations using an automated cell counter. Key biologic factor concentrations in the PRP preparations were also compared to those found in commercially available birth tissue-derived allograft products. The analyzed biologic factors included hepatocyte growth factor (HGF), interleukin-1 receptor antagonist (IL-1RA), fibroblast growth factor 2 (FGF-2), transforming growth factor-beta 3 (TGF-β3), tissue inhibitor of metalloprotease 1 (TIMP-1), alpha 2 macroglobulin (A2M), and proteoglycan 4 (PRG4).

Two commercially available PRP systems, Arthrex Angel and Apex Biologics PRP systems, were used according to the manufacturers’ recommendations [14,15]. All three subjects met the inclusion criteria and consented to the procedure. Fluids were encouraged in the first 24 hours before the blood draw. A total of 208 mL of whole blood was collected from the participants via venipuncture. A traditional tourniquet was applied to the participant’s arm, and the antecubital fossa was prepared in a sterile fashion. A 19-gauge butterfly needle was inserted into the median antecubital vein, and blood was aspirated into 60 mL syringes. After blood collection, the needle was removed, and an adhesive bandage was applied to the site.

For the Arthrex Angel System, a total of 104 mL of whole blood collected via venipuncture was mixed with 16 mL of anticoagulant citrate dextrose solution (ACDA) and transferred to the centrifuge. Of the 120 mL mixture, 60 mL was centrifuged at a 2% hematocrit concentration to create a leukocyte-poor (LP) PRP preparation, while the remaining 60 mL was centrifuged at a 7% hematocrit concentration to produce a leukocyte-rich (LR) PRP preparation, in accordance with the manufacturer’s recommendations. The Angel system, an automated PRP device, required no further manual preparation [16,17].

For the Apex Biologics System, a total of 50 mL of whole blood was mixed with 10 mL of ACDA and transferred to a centrifuge canister; this process was repeated twice for a total of 120 mL. Each 60 mL mixture was weighed on a scale to ensure equal weight for each canister. The blood was centrifuged for 10 minutes at 3,800 rpm. After centrifugation, the separated products were processed using the manual benchtop processing system. A piston was advanced through the APEX Biologics Benchtop Press, making contact with the base of the XCELL Concentration Device. This base was driven upward through the device, propelling the separated fractions into a collecting syringe attached to the top of the XCELL device. One canister was processed to create LP PRP, while the second canister was processed to create a LR PRP preparation [15].

Two milliliters of whole blood from each participant were set aside for baseline measurements, including hematocrit, leukocyte, and platelet concentrations, which were analyzed using a Horiba hematology automated cell counter in conjunction with the Stem Cell Partners Lab. Approximately 0.5 mL of each PRP sample was similarly analyzed with the Horiba cell counter to determine post-centrifugation hematocrit, leukocyte, and platelet concentrations. The remaining PRP samples were collected and cryopreserved for subsequent quantification of biological growth factors via enzyme-linked immunosorbent assay (ELISA) analysis.

The PRP preparations were analyzed for biological growth factor concentrations using ELISA. Additionally, commercially available birth tissue-derived allograft products were either purchased or received as donations for similar analysis. These products were stored according to the manufacturers’ recommendations, either at room temperature or frozen [18-21]. Details of the samples are provided in Table 1. To ensure sufficient volumes for testing, three separate lots of each product per manufacturer were analyzed. All sample quantities/volumes shown in Table 1 were adjusted to 2.5 mL using phosphate-buffered saline, a necessary step to accurately calculate concentrations. All protein analyses were performed at the UC Davis Center for Regenerative Cures. Commercial ELISA kits (R&D Systems) were utilized to detect and quantify the presence of biological factors.

**Table 1:** Summary of biologic products used in the study. PRP: platelet-rich plasma; LR: leukocyte-rich; LP: leukocyte-poor

#	Sample name	Description	Company	Product	Quantity	Lot/ID
1	Amniox	Particulate amniotic membrane and umbilical cord tissue product	Amniox Medical	Clarix Flo	25 mg	TGED20L004
2	Amniox	Particulate amniotic membrane and umbilical cord tissue product	Amniox Medical	Clarix Flo	25 mg	TGJK20L002
3	Amniox	Particulate placental tissue product	Amniox Medical	Neox Flo	50 mg	ZT19G004BTDS
4	Amniomatrix	Amniotic allograft suspension	Integra	Amniomatrix	1 mL	BF01670199
5	Amniomatrix	Amniotic allograft suspension	Integra	Amniomatrix	1 mL	BF01660088
6	Amniomatrix	Amniotic allograft suspension	Integra	Amniomatrix	0.5 mL + 0.5 mL	BF01640565 + BF01640566
7	Organicell	Amniotic fluid-derived extracellular vesicles	Organicell	Organicell Flow	1 mL	OC-HAF-012
8	Organicell	Amniotic fluid-derived extracellular vesicles	Organicell	Organicell Gold	1 mL	OC-HAF-042-G
9	Organicell	Amniotic fluid-derived extracellular vesicles	Organicell	Organicell Gold	1 mL	OC-HAF-049-G
10	Arthrex-LR	LR PRP	Arthrex	Angel	1 mL	A1-LR
11	Arthrex-LR	LR PRP	Arthrex	Angel	1 mL	A2-LR
12	Arthrex-LR	LR PRP	Arthrex	Angel	1 mL	A3-LR
13	Apex-LR	LR PRP	Apex Biologix	XCELL PRP	1 mL	B1-LR
14	Apex-LR	LR PRP	Apex Biologix	XCELL PRP	1 mL	B2-LR
15	Apex-LR	LR PRP	Apex Biologix	XCELL PRP	1 mL	B3-LR
16	Arthrex-LP	LP PRP	Arthrex	Angel	1 mL	A1-LP
17	Arthrex-LP	LP PRP	Arthrex	Angel	1 mL	A2-LP
18	Arthrex-LP	LP PRP	Arthrex	Angel	1 mL	A3-LP
19	Apex-LP	LP PRP	Apex Biologix	XCELL PRP	1 mL	B1-LP
20	Apex-LP	LP PRP	Apex Biologix	XCELL PRP	1 mL	B2-LP
21	Apex-LP	LP PRP	Apex Biologix	XCELL PRP	1 mL	B3-LP

Statistical analysis was performed using SPSS Version 28 (IBM Corp., Armonk, NY, USA). Analysis of variance for repeated measures with the Bonferroni test was conducted for post hoc comparisons. The confidence interval was set at 95%.

## Results

**Figure 1:**
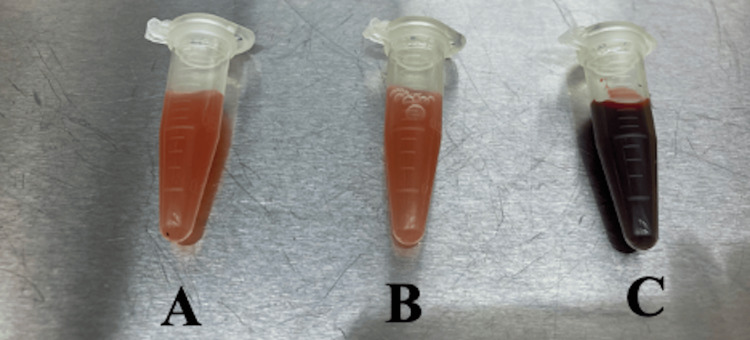
Vials of two PRP samples demonstrating anticipated color in samples compared to darker A3 leukocyte-poor PRP sample. PRP: platelet rich plasma

PRP characteristics are summarized in Table 2. In comparison to whole blood analysis, the PRP automated process was successful for all samples except A3. This particular sample exhibited a high number of red blood cells (RBCs), similar to that of whole blood, and a low platelet count, resulting in a platelet concentration factor of 0.8. Furthermore, the gross appearance of the sample was viscous and red, contrasting with the otherwise translucent and light yellow color of the other PRP samples (Figure 1). As expected, leukocyte-rich PRP samples demonstrated higher platelet and leukocyte concentrations and total counts.

**Table 2:** Summary of PRP characteristics obtained from three volunteers (1, 2, 3). Samples were subdivided into Arthrex Angel (A) and Apex Biologics (B) samples. The A3* leukocyte-poor sample is marked with an asterisk because it yielded a product that was darker compared to other samples (A1 and A2). MNC: mononuclear cells; PLT: platelets; PRP: platelet-rich plasma; RBCs: red blood cells; WBCs: white blood cells

	Arthrex Angel								Apex Biologics							
PRP Type	Leukocyte-poor				Leukocyte-rich				Leukocyte-poor				Leukocyte-rich			
Sample ID	A1	A2	A3^*^	Mean ± SD	A1	A2	A3	Mean ± SD	B1	B2	B3	Mean ± SD	B1	B2	B3	Mean ± SD
Volume (mL)	2.8	3.2	3.0	3.0 ± 0.2	3.0	3.2	3.4	3.2 ± 0.2	4.4	4.2	3.8	4.1 ± 0.3	5.6	5.0	4.8	5.1 ± 0.4
PLT concentration factor	8.3	7.9	0.8	5.7 ± 4.2	8.9	8.5	8.9	8.8 ± 0.2	4.8	10.2	4.9	6.6 ± 3.1	8.4	9.2	11.0	9.5 ± 1.3
PLT concentration (10^6^ cells/mL)	1,600	1,928	135	1,221 ± 955	1,712	2,088	1,467	1,756 ± 312	895	2,301	808	1,334 ± 838	1,569	2,081	1,815	1,822 ± 256
RBC concentration (10^9^ cells/mL)	0.11	0.16	6.45	2.24 ± 3.65	1.23	0.97	0.71	0.97 ± 0.26	0.06	0.13	0.02	0.07 ± 0.06	1.38	0.79	0.44	0.87 ± 0.47
WBC concentration (10^6^ cells/mL)	7.8	12.2	10.0	10.0 ± 2.2	26.2	33.1	18.4	25.9 ± 7.3	3.5	4.7	0.7	2.9 ± 2.1	27.4	38.3	21.3	29.0 ± 8.6
MNC concentration (10^6^ cells/mL)	7.4	10.3	2.5	6.7 ± 4.0	24.0	23.7	14.1	20.6 ± 5.6	3.2	4.0	0.5	2.6 ± 1.8	24.2	22.8	16.5	21.1 ± 4.1
Lymphocyte concentration (10^6^ cells/mL)	6.8	9.2	2.0	6.0 ± 3.6	20.3	19.0	11.5	16.9 ± 4.7	3.0	3.7	0.5	2.4 ± 1.7	21.8	19.4	14.2	18.4 ± 3.9
Monocytes (10^6^ cells/mL)	0.6	1.2	0.5	0.7 ± 0.4	3.7	4.7	2.6	3.1 ± 1.1	0.2	0.4	0.0	0.2 ± 0.2	2.4	3.5	2.3	2.7 ± 0.7
Granulocyte concentration (10^6^ cells/mL)	0.5	1.9	7.6	3.3 ± 3.8	2.2	9.4	4.3	5.3 ± 3.7	0.3	0.7	0.2	0.4 ± 0.3	3.3	15.5	4.8	7.8 ± 6.7
PLT total (10^6^ cells)	4,479	6,170	405	3,684 ± 2,963	5,136	6,680	4,989	5,602 ± 937	3,936	9,662	3,070	5,556 ± 3,582	8,786	10,405	8,712	9,301 ± 957
RBC total (10^9^ cells)	308	496	19,350	6,718 ± 10940	3,675	3,088	2,403	3,055 ± 637	242	546	57	282 ± 247	7,700	3,967	2,112	4,593 ± 2,846
WBC total (10^6^ cells)	21.8	38.9	30.0	30.2 ± 8.5	78.6	105.8	62.7	82.3 ± 21.8	15.2	19.5	2.5	12.4 ± 8.9	153.4	191.5	102.0	149.0 ± 44.9
MNC total (10^6^ cells)	20.6	33.0	7.4	20.3 ± 12.8	72.0	75.8	48.1	65.3 ± 15.1	13.9	16.8	1.9	10.9 ± 7.9	135.2	114.2	79.0	109.5 ± 28.4
Lymphocyte total (10^6^ cells)	18.9	29.3	6.0	18.1 ± 11.7	60.9	60.8	39.2	53.6 ± 12.5	13.0	15.3	1.9	10.1 ± 7.2	122.1	96.8	67.9	95.6 ± 27.1
Monocyte total (10^6^ cells)	1.7	3.7	1.4	2.2 ± 1.3	11.1	15.0	8.8	11.7 ± 3.1	0.9	1.5	0.0	0.8 ± 0.7	13.2	17.3	11.0	13.8 ± 3.2
Granulocyte total (10^6^ cells)	1.3	5.9	22.7	9.9 ± 11.2	6.6	29.9	14.6	17.0 ± 11.8	1.3	2.7	0.6	1.5 ± 1.1	18.2	77.3	23.0	39.5 ± 32.8

**Figure 2:**
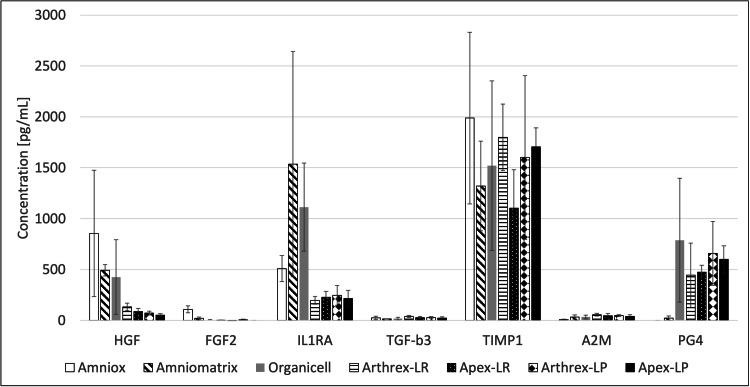
Bar graph summarizing the average concentrations of key biologic factors in all samples, with error bars depicting standard deviation. Using analysis of variance, the concentration of FGF2 was statistically significantly higher in the Amniox sample compared to all other groups (p < 0.001 for comparisons to all other samples). The concentration of A2M was statistically significantly higher in the Arthrex-LR sample compared to the Amniox sample (p = 0.048). Otherwise, no statistically significant differences were found among the samples. Data are represented as mean ± standard deviation. A2M: alpha-2-macroglobulin; FGF2: fibroblast growth factor 2; HGF: hepatocyte growth factor; IL-1RA: interleukin-1 receptor antagonist; LR: leukocyte-rich; LP: leukocyte-poor; PG4: proteoglycan 4; TIMP-1: tissue inhibitor of metalloproteinase 1

Table 3 and Figure 2 summarize the concentrations of key biologic factors. Significant within-group differences in biologic factor concentrations were observed, particularly for HGF in Amniox and Organicell, IL1RA in Amniomatrix, TIMP1 in Amniox, Organicell, and Arthrex-LP, and PRG4 in Organicell. Regarding between-sample differences, the concentration of FGF2 was statistically significantly higher in Amniox compared to all other groups (p < 0.001 for comparisons to all other samples). Additionally, the concentration of A2M was statistically significantly higher in the Arthrex-LR sample compared to the Amniox sample (p = 0.048). Otherwise, no significant statistical differences were found among the samples.

**Table 3:** Summary of the average concentrations ± standard deviation of key biologic factors in the samples. Data are expressed in pg/mL. The p-value represents the results of analysis of variance. Statistically significant differences in the average concentrations were observed for HGF, FGF2, IL-1RA, and PG4. Post hoc comparisons showed that the concentration of FGF2 was statistically significantly higher in the Amniox sample compared to all other groups (p < 0.001 for comparisons to all other samples). The concentration of A2M was statistically significantly higher in the Arthrex-LR sample compared to the Amniox sample (p = 0.048). A2M: alpha-2-macroglobulin; FGF2: fibroblast growth factor 2; HGF: hepatocyte growth factor; IL-1RA: interleukin-1 receptor antagonist; LR: leukocyte-rich; LP: leukocyte-poor; PG4: proteoglycan 4; SD: standard deviation; TGF-β3: transforming growth factor beta 3; TIMP-1: tissue inhibitor of metalloproteinase 1

	Amniox	Amniomatrix	Organicell	Organicell	Arthrex-LR	Apex-LR	Arthrex-LP	Apex-LP	P-value
HGF	855 ± 620	493 ± 56	426	426 ± 366	133 ± 38	87 ± 31	73 ± 19	53 ± 13	0.022
FGF2	109 ± 34	21 ± 12	3	3 ± 6	2 ± 3	0 ± 0	6 ± 8	0 ± 0	<0.001
IL1RA	509 ± 129	1,537 ± 1,107	1,113	1,113 ± 432	198 ± 38	227 ± 58	247 ± 98	215 ± 82	0.013
TGFb3	25 ± 17	14 ± 2	18	18 ± 15	39 ± 12	28 ± 12	30 ± 8	24 ± 11	0.227
TIMP1	1,989 ± 843	1,321 ± 441	1,521	1,521 ± 832	1,799 ± 328	1,104 ± 375	1,601 ± 805	1,707 ± 186	0.632
A2M	8 ± 5	33 ± 21	33	33 ± 20	57 ± 14	46 ± 22	47 ± 10	40 ± 16	0.054
PG4	0 ± 0	23 ± 20	788	788 ± 609	445 ± 315	475 ± 67	659 ± 315	600 ± 134	0.030

**Figure 3:**
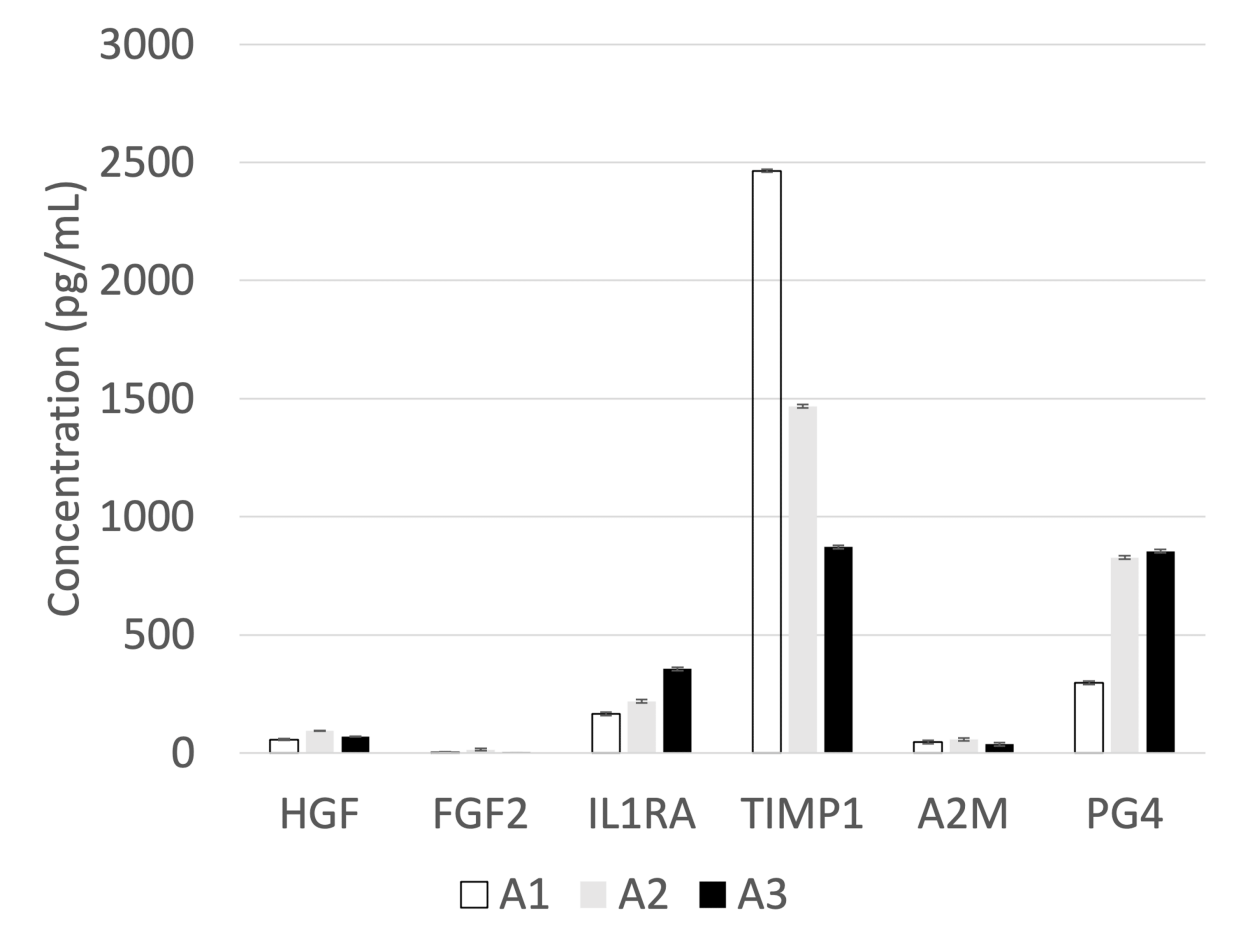
Bar graph summarizing the average concentrations of key biologic factors in Apex-LP samples. The A3 sample had a platelet concentration factor of only 0.8, compared to 8.3 for A1 and 7.9 for A2. Despite the significant differences in platelet concentration factors, most of the biologic factors among the Apex-LP samples were comparable, with the exception of TIMP1, which showed a notable difference. Error bar shows standard deviations. A2M: alpha-2-macroglobulin; FGF2: fibroblast growth factor 2; HGF: hepatocyte growth factor; IL-1RA: interleukin-1 receptor antagonist; PG4: proteoglycan 4; TIMP-1: tissue inhibitor of metalloproteinase 1

Figure 3 presents an in-depth analysis of biologic factors comparing the Apex-LP samples. In the third Apex-LP sample (A3), where the platelet concentration factor was only 0.8, compared to 8.3 for A1 and 7.9 for A2, the only biologic factor that showed a remarkably reduced concentration was TIMP-1. The concentrations of TIMP-1 were 2,465 pg/mL for A1, 1,467 pg/mL for A2, and 872 pg/mL for A3.

## Discussion

This is the first study to compare the biologic factors among commercially available allograft tissues and PRP. The results showed that Amniox, a particulate amniotic product, contained a higher concentration of FGF2 compared to other samples. Additionally, the concentration of A2M in the Arthrex LR PRP product was higher compared to the Amniox product. Otherwise, the allograft products and PRP contained comparable levels of key biologic factors. Furthermore, despite the remarkable differences in platelet concentration factors among the three Apex-LP PRP samples, the only biologic factor affected was TIMP1.

The biologic factors quantified in this study were selected based on their believed roles in modulating the inflammatory processes involved in tissue injury and recovery. HGF and IL1RA have anti-inflammatory effects, with the potential to reduce pain and improve function in painful joints and musculoskeletal tissues [22]. FGF-2 has anabolic effects, with the potential to stimulate soft tissue proliferation and healing [23]. TIMP1 and A2M may help eliminate degenerative proteins from joints, with the potential to improve joint function [24]. Finally, PG4 may contribute to joint lubrication, further supporting the healing process [25].

This study highlights substantial variability in the levels of key biologic factors in commercially available birth tissue-derived products. Notably, the differences in protein levels among lots from the same product were significant. These findings mirror the variability observed in biologic proteins between lots of the same product in our amniotic fluid study [12]. Given the considerable inconsistency of therapeutically relevant proteins between product lots within the same product line, the variability in the content of biologic factors could potentially impact expected therapeutic outcomes.

Several factors could contribute to these variations. Davies et al. cite the lack of consensus regarding cord anatomy and the definitions of various regions/zones of the cord [26]. Although the same lot typically corresponds to a product coming from the same donor, it is possible that variations in anatomic composition within the same amnion could occur depending on the harvest site. Additional variables to consider include the procedural methods of tissue isolation, preservation, and formulation, including the particularization process [1,27-29].

PRP demonstrated less variability between donors, suggesting it may be a more consistent product. Notably, there was no significant difference between LP and LR PRP preparations. This was an unexpected finding, as a higher amount of key biologic factors was anticipated in the LR preparation due to its higher platelet count, which typically correlates with more biologic factors. Furthermore, when comparing the three samples from Apex-LP PRP, despite significant differences in platelet concentration factors, all biologic factors except TIMP1 showed comparable concentrations. This suggests the need for further investigation into the concentration of these key biologic factors.

The concentrations of biologic factors and proteins tested, while important, represent only a few of the many factors that constitute the dynamic, environmental milieu. Their production and activity can vary depending on their environment and other processes. Additionally, some of the allografts tested may rely on other properties for their primary mechanism of action. For example, exosomes may primarily rely on microRNA proteins, or amnion may depend on extracellular matrix properties for scaffolding. Therefore, quantifying growth factors and cytokine concentrations alone may not provide a comprehensive characterization of these biological products.

This study has several limitations. Its applicability is restricted by the small number of allografts and PRP samples, which were constrained by financial limitations and the availability of allograft products. Additionally, the third Apex LP-PRP sample (A3) exhibited a reduced platelet concentration factor. Nevertheless, the data from this sample were included in the analysis, as the study’s primary objective was to quantify the concentrations of biological factors and examine how variability in preparation methods could influence these results. It is also important to note that, in the United States, birth tissue-derived products are regulated under the Human Cells, Tissues, and Cellular and Tissue-Based Products guidelines issued by the Food and Drug Administration. Currently, no birth tissue-derived allografts should be administered to patients outside of an investigational new drug study.

## Conclusions

This study provides a comparative analysis of key biologic factors in birth tissue-derived allografts and PRP preparations. Allograft products generally exhibited similar concentrations of key biologic factors, with the exception of FGF2, which was significantly higher in the particulate amniotic membrane product. However, notable variability in biologic factor concentrations among different product lots raises concerns regarding the reproducibility and consistency of these products, which could impact therapeutic outcomes. In contrast, PRP preparations demonstrated greater consistency, with comparable levels of biologic factors observed between LR and LP preparations. These findings emphasize the need for greater standardization in the processing and manufacturing of allograft products to ensure reproducible therapeutic content. Further research is warranted to explore these mechanisms and assess the clinical relevance of variability in biologic factor concentrations.
